# Relative age effect in high-skill labor markets: evidence from the NBA

**DOI:** 10.3389/fspor.2026.1787778

**Published:** 2026-05-07

**Authors:** Simcha Avugos, Yossi Haleva

**Affiliations:** School of Human Movement and Sport Sciences, Levinsky-Wingate Academic Center – Wingate Campus, Netanya, Israel

**Keywords:** basketball, date of birth, relative age effect, sport labor market, talent development

## Abstract

This study examined whether the relative age effect (RAE) exists among NBA players and whether relatively younger athletes (Q4-born) display compensatory qualities, such as greater height, weight, or a higher likelihood of playing Center. Birth dates, anthropometric characteristics, playing positions, and draft outcomes of all players in the 2024 season (*n* = 537) were analyzed. Chi-square tests and regression analyses revealed no significant skew toward earlier birth months. Likewise, late-born players were not taller, heavier, more likely to play Centers, or differently represented in draft outcomes compared to peers. These findings suggest that while a survivor effect may influence earlier stages of athletic development, it does not extend to adult labor market outcomes in the NBA. The results highlight implications for coaches and scouts, suggesting that attention to late-born athletes can improve talent utilization and reduce potential losses in the wider sport system, both at youth level and within feeder systems to the professional league.

## 1 Introduction

This study examines whether birth dates among NBA players reflect a relative age effect (RAE), and whether RAE influences entry into the league differently for drafted vs. undrafted players. Additionally, regardless of whether RAE is observed, the study explores whether late-born players who reach the top exhibit compensatory traits, such as height, weight, or playing position. By investigating these patterns, the study aims to understand whether relative age continues to shape professional basketball outcomes or whether other physical and positional characteristics dominate selection at the elite level.

Although the study focuses on NBA rosters, RAE is typically understood as the downstream result of selection biases in youth development systems. A skewed distribution of birth months may reflect long-term effects of youth sports selection, where athletes born earlier in the selection year (e.g., January-March) are more likely to be identified as talented compared to those born later (e.g., October-December). Early-born athletes often have better motor skills (balance, coordination, speed, and strength), more advanced physical development (height, muscle mass), and greater aerobic fitness (1). These athletes therefore receive further development opportunities, including access to better coaching and competition (2, 3). Such early advantages may have persistent effects on who reaches the top of an elite labor market.

Research on RAE in international youth and professional contexts shows mixed results. Specifically in basketball, some studies at youth or early development stages show strong RAE patterns (e.g., 4–7), while others – especially at elite youth and senior international levels, and among female athletes – show little or no effect (e.g., 8, 9). Despite being the world's top basketball league, the NBA received limited attention in RAE research. Based on the literature we are aware of, Leite et al. (10) reported an over-representation of early born (Q1/Q2) players among NBA draftees, whereas Steingröver et al. (11) found no significant RAE effect on career length, likely because initial selection advantages at entry do not necessarily translate into longer professional careers.

RAE has been particularly evident in team sports requiring high contact and strength (e.g., 12, 13), but it has also been observed in non-contact and individual sports, such as tennis, swimming, and athletics (e.g., 14, 15). In several studies, RAE appears reversed, with athletes born later in the selection year outperforming older peers over time (e.g., 16, 17).

Few studies examine RAE using a clear labor market/economic perspective, such as salary or career outcomes. For example, Ashworth and Heyndels (18) investigated how RAE influences earnings within the German soccer league, and Deaner et al. (19) reported a type of “underdog effect” among relatively younger hockey draftees in the NHL, who often have longer and more successful careers. Outside of sports, limited work has explored RAE in broader career contexts, including CEOs of S&P 500 companies (20), U.S. legislators (21), Finnish parliament members (22), and U.K. Nobel laureates (23), suggesting that relative age may influence success across high-skill domains.

This paper contributes to the literature in several ways. First, empirical identification of RAE is important because its existence implies systematic bias in selection process – a form of labor market inefficiency. Providing that such a bias exists in elite sports speaks to issues of fairness, resource allocation, and human capital misallocation (24, 25). Second, if RAE is strong, it may suggest that current youth cut-off dates systematically disadvantage certain birth cohorts, leading to under-utilization of talent – a form of market failure. Third, even if no classical RAE is found at the professional level, late-born players who reach the NBA may be taller, or heavier on average, and may more often occupy positions (e.g., Centers) that rely on size rather than relative age in youth leagues. This reflects a form of self-selection or compensatory advantage, providing insights into how elite, high-skill labor markets filter exceptional ability rather than average talent.

## 2 Method

### 2.1 Data collection

The sample included 537 players from all 30 teams during the 2024 season. Data were collected from the official NBA database (https://nba.com/players) between December 22, 2024 and January 15, 2025. For each player, we collected data on name, team, date of birth, country, height, weight, playing position, and draft details.

### 2.2 Data analysis

Statistical analyses were conducted using IBM SPSS statistics software (version 19.0; IBM SPSS, Armonk, NY, USA). Statistical significance was set at *p* ≤ .05. Players’ dates of birth were grouped into four quartiles (Q1–Q4) based on month of birth. Two alternative cut-off dates – January 1 and September 1 – were used do define quartiles, reflecting school-entry systems in the U.S. and other countries.

Chi-square tests were used to determine whether the distribution of birth dates differed from a uniform distribution in the full sample. For American players, the distribution was also compared with official national live birth statistics (retrieved August 31, 2025, from https://www.cdc.gov). Cramér's V was calculated to assess the strength of association between birth quarter and player representation in the league. Odds ratios (OR) with 95% confidence intervals (CI) were calculated comparing Q1 and Q4.

Chi-square test of independence was also used to examine whether the distribution of birth quarters differed between drafted and undrafted players. ORs of being drafted for Q4 vs. Q1–Q3, and separately vs. Q1 and Q2, were calculated.

We examined whether players’ anthropometric characteristics and playing positions differed by birth quarter (BQ). Birth quarter was coded as a categorical variable with four levels (Q1–Q4), with Q4 (relatively youngest players) as the reference category. To test whether height and weight varied by birth quarter, two separate linear regression models were conducted with height (cm) and weight (kg) as dependent variables and BQ as the independent variable. Regression coefficients for Q1, Q2, and Q3 (dummy variables) indicate the mean difference relative to Q4.

To examine whether Q4 players were more likely to be centers, a 2 × 2 contingency table was created comparing Q4 vs. Q1–Q3 and Center vs. Non-Center players. Players listed as both center and another position (e.g., center-forward; *n* = 50) were classified as centers for the analysis. Chi-square test of independence was used to evaluate this association, and odds ratios (OR) with 95% confidence intervals (CI) were calculated to quantify the likelihood of Q4 players being centers.

## 3 Results

### 3.1 Background data

Among the 537 NBA players, most were American (*n* = 409, 76.2%), and 121 (22.5%) were undrafted, of which 102 were Americans. The average age was 26.17 years (SD = 4.34), ranging from 18.9 (Ulrich Chomche, Cameroon; Toronto Raptors) to 39.9 years (LeBron James, USA; Los Angeles Lakers). The oldest player was born in December 1984, the youngest in December 2005.

Players’ average height was 199.55 cm (SD = 7.99), ranging from 172.7 (Yuki Kawamura, Japan; Memphis Grizzlies) to 223.5 cm (Zach Edey, Canada; Memphis Grizzlies). Average weight was 97.45 kg (SD = 10.67), ranging from 72 (Yuki Kawamura) to 138 kg (Zach Edey).

Non-American players were significantly taller (M = 204.21 cm, SD = 7.84) than American players (M = 198.09 cm, SD = 7.46; *p* < .05). A height ≥ 2.05 m was observed in 64 of 128 non-American players vs. 87 of 409 American players. Age did not differ by nationality (M = 26.18, SD = 4.48 vs. M = 26.17, SD = 4.30; *p* = .98). Year-of-birth distribution for all players was: 1984–1989, *n* = 25; 1990–1999, *n* = 259; and 2000–2005, *n* = 253.

### 3.2 Empirical RAE analysis

Assuming equal birth distribution, the year was divided into four quarters, adjusted for days per month: Q1 (Jan–Mar) 24.7%, Q2 (Apr–Jun) 24.9%, Q3 (Jul–Sep) 25.2%, and Q4 (Oct–Dec) 25.2%. This served as the expected distribution for the *χ*^2^ tests.

Table 1 shows the number and percentage of players by quartile. A peak was observed in Q3 for the total sample (26.63%) and U.S. players (27.63%), but not for non-U.S. players (23.44%). The highest number of births was in July (*n* = 51) and the lowest in December (*n* = 33), though this pattern varied by nationality.

**Table 1:** Distribution of players’ birth month by nationality and statistical analyses.

Q distribution	Quartile and number and percentage of NBA players (Cut-off date: January 1)								
	Jan-Mar 24.7%	Apr-Jun 24.9%	Jul-Sep 25.2%	Oct-Dec 25.2%	Total	*χ*^2^ (df = 3)	*p*	Cramér's V	Q1 vs. Q4: OR (95% CI)
All players (%)	133 (24.77)	134 (24.95)	143 (26.63)	127 (23.65)	537	0.95	.81	0.03	1.07 (0.761–1.506)
Expected	132.41	133.88	135.35	135.35					*p* = .67
U.S. players (%)	103 (25.18)	92 (22.49)	113 (27.63)	101 (24.69)	409	2.02	.57	0.05	1.04 (0.707–1.537)
Expected	100.85	101.97	103.09	103.09					*p* = .84
Other players (%)	30 (23.44)	42 (32.81)	30 (23.44)	26 (20.31)	128	4.64	.20	0.13	1.18 (0.575–2.420)
Expected	31.56	31.91	32.26	32.26					*p* = .65

Q distribution	Quartile and number and percentage of NBA players (Cut-off date: September 1)								
	Sep-Nov 24.9%	Dec-Feb 24.7%	Mar-May 25.2%	Jun-Aug 25.2%	Total	*χ*^2^ (df = 3)	*p*	Cramér's V	Q1 vs. Q4: OR (95% CI)
All players (%)	141 (26.26)	116 (21.60)	138 (25.70)	142 (26.44)	537	2.79	.43	0.05	1.00 (0.719–1.402)
Expected	133.88	132.41	135.35	135.35					*p* = 1.00
U.S. players (%)	117 (28.61)	88 (21.52)	99 (24.21)	105 (25.67)	409	4.05	.26	0.07	1.13 (0.770–1.647)
Expected	101.97	100.85	103.09	103.09					*p* = .53
Other players (%)	24 (18.75)	28 (21.88)	39 (30.47)	37 (28.91)	128	4.47	.22	0.13	0.66 (0.323–1.333)
Expected	31.91	31.56	32.26	32.26					*p* = .25

OR, odds ratio; CI, confidence interval.

No significant deviations from the expected distribution were observed for the total sample [*n* = 537, *χ*^2^(df = 3) = 0.95, *p* = .81], U.S. players [*n* = 409, *χ*^2^(df = 3) = 2.02, *p* = .57], or non-U.S. players [*n* = 128, *χ*^2^(df = 3) = 4.64, *p* = .20]. Effect sizes were small (Cramér's V = 0.03–0.13). Odds ratios for Q1 vs. Q4 were close to 1 with wide confidence intervals, and *p*-values were all high (.65–.84), indicating no meaningful RAE.

Across all groups, Q4-born players were slightly underrepresented relative to Q2 and Q3, but none of these differences reached significance (total: *p* = 0.71, 0.49; U.S.: *p* = 0.68, 0.57; non-U.S.: *p* = 0.16, 0.70).

Using a September 1 cut-off produced similar results: no significant differences (all *p* > .20; Cramér's V ≤ 0.13). The confidence intervals and high *p*-values (.25–1.00) are consistent with the absence of a meaningful Q1 vs. Q4 effect. Q4-born players did not differ significantly from Q2 or Q3 in any group (total: *p* = 0.30 and 0.87; U.S.: *p* = 0.44 and 0.76; non-U.S.: *p* = 0.46, 0.87).

To summarize, the results were consistent across both cut-off dates, with no significant differences between Q4 and other quartiles across all groups. Non-U.S. players showed a mild but non-significant Q2 overrepresentation relative to Q4 under the January 1 cut-off (OR = 1.63, *p* = 0.16), which disappeared under the September 1 cut-off (OR = 0.77, *p* = 0.46). Among U.S. players and in the total sample, odds ratios remained close to 1 under both cut-offs, confirming no meaningful RAE.

In the next phase, we analyzed RAE relative to normative population values.

NBA players originate from many countries with varying birth patterns. Because most non-U.S. national groups were too small for meaningful comparison, birth dates were tested against national birth data only for U.S. players (*n* = 409), born between 1984 (LeBron James) and 2005 (Ulrich Chomche). U.S. live birth data were retrieved from the CDC Natality Series (https://www.cdc.gov/nchs/products/vsus.htm) (26).

As shown in Table 2, *χ*^2^ tests revealed no significant deviation from U.S. birth rates [January 1 cut-off: *χ*^2^(df = 3) = 1.30, *p* = .73; September 1 cut-off: *χ*^2^(df = 3) = 2.99, *p* = .39]. Effect sizes were very small (Cramér's V = 0.04 and 0.06). Odds ratios for Q1 vs. Q4 were close to 1, and the wide confidence intervals with high *p*-values (.81 and.47) confirm the absence of a meaningful RAE.

**Table 2:** Distribution of players’ birth month compared to national live births in the U.S. and statistical analyses.

Q distribution [U.S. birth data]	Quartile and number and percentage of NBA players (Cut-off date: January 1)								
	Jan-Mar 24.0%	Apr-Jun 24.8%	Jul-Sep 26.4%	Oct-Dec 24.8%	Total	*χ*^2^ (df = 3)	*p*	Cramér's V	Q1 vs. Q4: OR (95% CI)
U.S. players (%)	103 (25.18)	92 (22.49)	113 (27.63)	101 (24.69)	409	1.30	.73	0.04	1.05 (0.712–1.554)
Expected	98.33	101.26	107.99	101.43					*p* = .81

Q distribution [U.S. birth data]	Quartile and number and percentage of NBA players (Cut-off date: September 1)								
	Sep-Nov 25.2%	Dec-Feb 24.0%	Mar-May 24.8%	Jun-Aug 26.0%	Total	*χ*^2^ (df = 3)	*p*	Cramér's V	Q1 vs. Q4: OR (95% CI)
U.S. players (%)	117 (28.61)	88 (21.52)	99 (24.21)	105 (25.67)	409	2.99	.39	0.06	1.15 (0.788–1.678)
Expected	103.08	98.07	101.48	106.36					*p* = .47

OR, odds ratio; CI, confidence interval.

**Figure 1:**
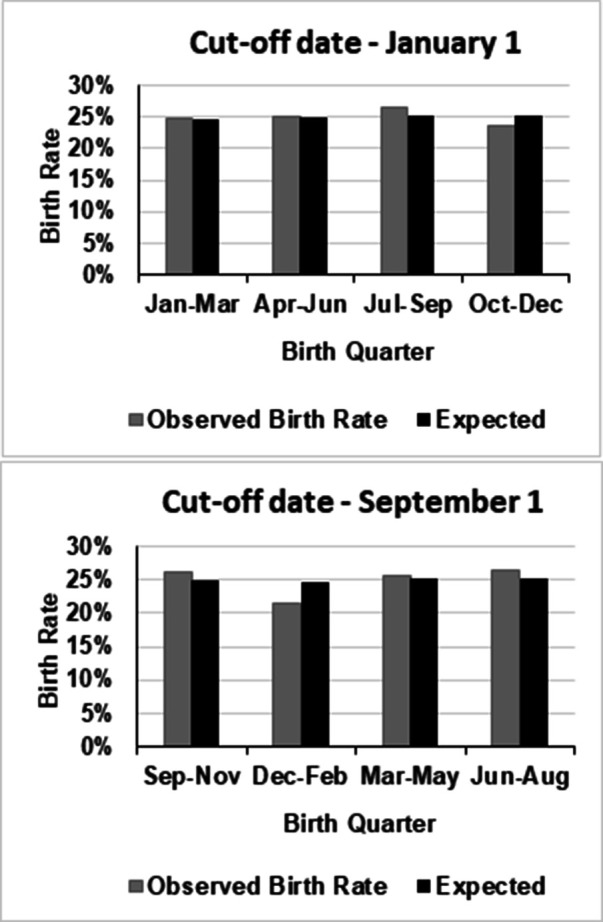
Comparison of NBA players’ birth rates with the expected uniform distribution.

**Figure 2:**
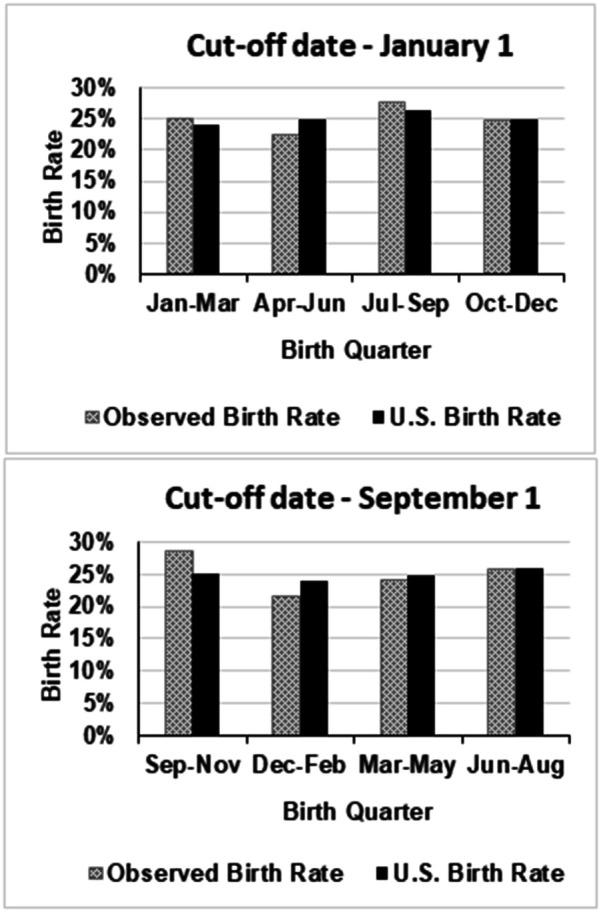
Comparison of NBA players’ birth rates with those of the general U.S. population.

Figures 1, 2 illustrate the distribution of birth months compared to a uniform distribution and to the U.S. live birth data, respectively.

### 3.3 Drafted vs. undrafted players by birth quarter

We tested whether drafted vs. undrafted players (*n* = 416 vs. 121, respectively) differ by birth quarter. The *χ*^2^ test showed no significant difference across birth quarters [*χ*^2^(df = 3) = 4.46, *p* = .22]. When comparing Q4-born players with Q1–Q3 combined, no significant association was found [*χ*^2^(df = 1) = 1.09, *p* = .0.30]. Odds ratio (OR < 1) indicated that Q4-born players were slightly less likely to be drafted compared to Q1–Q3 (OR = 0.78, 95% CI = 0.504–1.202), but this effect was not statistically significant (*p* = .30).

Direct comparisons confirmed this pattern. Players born in Q1 had higher odds of being drafted than those born in Q4 (OR = 1.26, 95% CI = 0.711–2.230), but the difference was not significant (*p* = .43). Similarly, Q2-born players had nearly identical odds of being drafted compared to Q4 (OR = 0.96; CI = 0.551–1.657, *p* = .87), reinforcing the absence of systematic differences. In fact, the odds of being drafted for Q2-born players were 4% lower than for Q4-born players.

Together, these results indicate that Q4-born players were not systematically disadvantaged in draft outcomes. By the time that players reach the NBA, those born later in the selection year appear to have overcome early disadvantages, likely because only individuals with exceptional talent, size, or perseverance progress to this level.

To explore this further, we compared body size between drafted and undrafted players. Drafted players were, on average, taller (M = 199.97 cm, SD = 7.94) than undrafted players (M = 198.10 cm, SD = 8.02), a statistically significant but small difference (*t* = 2.28, *p* = .02; Cohen's d = 0.24). For weight, drafted players (M = 97.93 kg, SD = 10.68) were slightly heavier than undrafted players (M = 95.79 kg, SD = 10.50), but this difference (∼2.15 kg) did not reach conventional significance (*t* = 1.95, *p* = .052; Cohen's d = 0.20).

### 3.4 Birth quarter effects on height, weight, and playing position

#### 3.4.1 Height

The regression model explained less than 1% of the variance in players’ height (R^2^ = 0.0075). After adjusting for the number of predictors, the exploratory power was essentially zero (adjusted R^2^ = 0.0019). In practical terms, this means that birth quarter does not account for meaningful differences in player height. The overall regression was not statistically significant (F = 1.34, *p* = .26).

The intercept (200.24 cm) represents the average height of Q4-born players. Relative to this group, Q1-born players were on average 1.57 cm shorter (coefficient = −1.57, *p* = .11). Although this difference might appear noticeable in basketball context, the lack of statistical significance and wide confidence interval (95% CI includes 0) suggest it is likely due to chance. Q2-born players showed virtually no difference compared to Q4 (coefficient = 0.003, *p*∼1.00), while Q3-born players were 1.14 cm shorter (coefficient = −1.14, *p* = .24). Again, the confidence intervals for these estimates included 0.

Taken together, the results indicate that birth quarter does not significantly predict NBA players’ height. Any apparent differences, such as Q1 players being slightly shorter, are minor and not statistically robust.

#### 3.4.2 Weight

The regression model explained less than 1% of the variance in players’ weight (R^2^ = 0.0077; adjusted R^2^ = 0.0021). This is an extremely small proportion, and the overall regression was not statistically significant (F = 1.39, *p* = .25). Thus, birth quarter as a whole does not explain variation in player weight.

The intercept (98.90 kg) represents the mean weight of Q4-born players. Compared to Q4, Q1-born players were on average 2.5 kg lighter (coefficient = −2.50, *p* ∼.06). Although this difference approaches marginal significance, the confidence interval includes 0, which suggests it is not reliable. Q2-born players were 1.17 kg lighter (coefficient = −1.17, *p* = .37), and Q3-born players were 2.03 kg lighter (coefficient = −2.03, *p* = .12). These effects were small and statistically non-significant.

In short, birth quarter has virtually no explanatory power for weight among NBA players. While Q1– and Q3-born players appear slightly lighter on average, these differences are small, not statistically significant, and unlikely to have practical meaning.

#### 3.4.3 Playing position

We first tested whether Centers (*n* = 102) and Non-Centers (*n* = 435) differed by birth quarter. The *χ*^2^ test indicated no significant association between birth quarter and playing position [*χ*^2^(df = 3) = 4.48, *p* = .21]. In other words, the distribution of Centers and Non-Centers was similar across quarters.

Next, we compared Q4-born players with those born in Q1–Q3 combined. The *χ*^2^ test again showed no significant difference in playing position [*χ*^2^(df = 1) = 0.0095, *p* = .92]. The observed and expected counts were nearly identical. The odds ratio for being a Center if born in Q4 (vs. Q1–Q3) was 1.06, suggesting only 6% higher odds. However, the 95% CI (0.641–1.752) and *p*-value (.82) indicate that this effect is not statistically significant and likely due to chance. Thus, there is no evidence that Q4-born players are more likely to be Centers.

## 4 Discussion

### 4.1 General discussion

This study examined whether RAE exists among NBA players and whether late-born athletes display distinct physical or positional attributes that might indicate a “survivor effect.” Contrary to expectations derived from the youth sport literature (e.g., 6, 27, 28), our findings did not support the presence of RAE at the professional level. The distribution of birth dates among NBA players did not differ significantly from chance, and Q4-born players were not taller, heavier, or more frequently represented among Centers (typically the tallest and strongest players) compared to their peers. Draft outcomes were also unrelated to birth quarter.

Relatively few studies have examined the relationship between body size and RAE in basketball, and most have focused on youth or adolescent samples, where early selection may be influenced by temporary physical advantages (e.g., 6, 29, 30). These studies suggest that late-born players who succeed in youth cohorts often display compensatory physical traits (e.g., height, weight, arm span, hand length) or performance capacities (e.g., sprint and jump ability). In addition, positions requiring greater height and body mass, such as Center, may be disproportionately allocated to early-born players during developmental stages, reinforcing selection bias (31). By contrast, our results align with research on elite adult athletes showing that early age-related differences observed in youth systems are no longer evident (e.g., 32–34).

At earlier stages of talent identification, Q4 players may face disadvantages relative to their older peers, and prior research suggests that some overcome these through physical development (e.g., being tall or strong despite their younger age), skill acquisition, resilience, or tactical intelligence. However, because the present study does not examine earlier developmental stages, we cannot determine how these processes unfold. The NBA represents the end-point of an exceptionally selective process, where only the most talented players remain, regardless of birth quarter. Thus, although a survivor effect may operate in youth systems, no relative age differences are detectable in adult labor market outcomes at the NBA level.

Extensive evidence demonstrates the presence of RAE in both U.S. and international basketball development systems, where age grouping shapes access to elite coaching, competition, and advancement opportunities. In U.S. collegiate basketball, patterns consistent with RAE have been observed in NCAA Division I competition, with relatively older athletes more commonly represented than expected relative to population norms, and national high school enrollment data confirm that this cannot be explained solely by birth distribution in the general population (35). Internationally, RAE is well documented in youth basketball. Analyses of elite under-18 European competitions show that a large majority of players (S1 = 67%; S2 = 68%) were born in the first half of the selection year compared with a uniform birth distribution (31), and data from over 150,000 licensed youth male players in France reveal significant early-born advantages across age groups (6). Cross-sport analyses including youth basketball in Germany and Austria for athletes aged 10–18 also demonstrated significant RAE effects (36). Systematic reviews further confirm that relatively older athletes are consistently over-represented in competitive U14–U18 categories compared with later-born peers (2, 37).

These findings indicate that RAE is a robust feature of feeder systems across contexts. Evidence from other sports suggests that the persistence and magnitude of RAE vary by sport, gender, and competitive level [e.g., ice hockey (38); handball (39); volleyball (40)]. For example, strong RAE has been reported in youth soccer, while in women's soccer it appears at younger ages and in men's soccer it can persist into senior international tournaments (41). Similarly, studies of youth athletes across multiple sports indicate that RAE generally decreases as age and competition levels increase (2, 42). However, the absence of RAE at the NBA level suggests that these early advantages may be attenuated or filtered out through successive stages of talent selection. Only athletes with exceptional and durable performance capacities reach the professional stage. In this sense, the NBA reflects the outcome of cumulative selection rather than the absence of early bias.

From a labor market perspective, the NBA can be understood as an extreme case of a high-skill, tournament-based labor market (43), in which rewards are highly concentrated among top performers (All-Stars, starters, and high draft picks) and determined by relative ranking rather than absolute differences in output. In such superstar markets, characterized by global scouting and intense competition, selection and compensation are closely tied to ranking, which is strongly influenced by observable productivity (25). Within this performance-driven framework, Human Capital Theory (44) helps explain why sustained skill accumulation – through sport-specific skills, physical development, and tactical knowledge – ultimately outweighs early relative age advantages, which temporarily inflate perceived potential (due to maturity-related differences) in youth systems. The NBA draft and free-agent market therefore operate as corrective filters, reallocating opportunities based on realized productivity rather than early developmental advantages. The lack of evidence of compensatory overrepresentation among Q4-born players in the present study suggests that those who reach the NBA are not systematically different in measurable ways from their peers.

Economic analyses of the NBA indicate that draft position and early selection status influence playing time, contract opportunities, and long-term earnings (45, 46), showing that early career advantages can generate durable labor returns. Research examining NBA-drafted players has identified relative age and birthplace effects at the point of draft selection, yet has found no significant differences in subsequent career performance (e.g., analysis of 1990–2019 draft cohorts; 10). Additionally, NBA-specific evidence shows that initial age advantages have minimal impact on career length (11). However, direct studies linking RAE to salaries or in-game performance in the NBA remain scarce (47, 48). Against this background, the absence of a league-wide RAE in the present study indicates that age-based advantages do not systematically structure professional attainment. Rather, by the final stage of the hierarchy, career success appears to reflect accumulated human capital and sustained performance more than early maturation differences or arbitrary age cut-offs.

The absence of RAE at the NBA level has practical implications for talent development. At the youth level, coaches and scouts should avoid overvaluing relative maturity when evaluating athletes, as late-born players can achieve comparable performance if given equal opportunities. Interventions such as bio-banding (grouping athletes by biological maturity rather than chronological age), rotating cut-off dates for age groups, or targeted support programs for younger athletes may help reduce the loss of potential elite players.

At the professional level, the findings suggest that by draft eligibility, relative age is unlikely to meaningfully predict physical profile or positional allocation. Scouting departments should therefore focus on measurable performance indicators and developmental trajectories rather than residual maturity advantages. Professional franchises that invest in long-term scouting networks – including international pathways and G league development – can further reduce relative age biases by broadening the talent pool and allowing late developers more time to mature through multiple entry pathways. Thus, while the NBA draft itself appears largely merit-based at the entry point, earlier developmental systems may still benefit from targeted structural adjustments to reduce relative age bias.

### 4.2 Limitations and future research

This study has several limitations. First, the analysis is based on a single NBA season (2024). Although the dataset covers the full league, the cross-sectional design does not allow assessment of temporal stability. In a dynamic labor market shaped by draft cycles, international recruitment, and cohort turnover, future research should examine data across multiple seasons to determine whether the absence of RAE reflects a stable structural pattern or short-term fluctuations. Nevertheless, including the entire population of active players provides a valid snapshot of the current elite labor market.

Second, the study includes only athletes who successfully reached the NBA and cannot capture those filtered out earlier in the system due to relative age or other selection biases. From a labor market and human capital perspective, the NBA represents the final stage of selection, and earlier differences in investment, opportunity, and skill development are not directly observed. Future studies should include NBA junior age groups to assess RAE prior to entry into the professional league.

Third, although the full sample includes all NBA players in the 2024 season, statistical power decreases in smaller subgroups (e.g., Centers only, *n* = 102; non-U.S. players, *n* = 128). Small effects could therefore go undetected, raising the possibility of Type II error. However, the observed effect sizes were consistently trivial (Cramér's V ≤ 0.13; ORs close to 1), suggesting that even if detectable in a larger multi-season sample, any underlying RAE at the NBA level would likely be practically negligible. This supports the interpretation that the NBA reflects the terminal stage of talent selection, where early advantages from relative age are largely filtered out.

Finally, the analysis focused primarily on observable characteristics, such as height, weight, and positional roles. Other important physical measures relevant in the NBA (e.g., wingspan, and vertical jump ability), as well as psychological, tactical, and motivational attributes, were beyond the scope of the available data. Future research should incorporate these dimensions and extend the framework to other professional leagues, women's basketball, and sports with different developmental structures.

## 5 Conclusion

While relative age may influence early opportunities in youth sport, the NBA labor market ultimately erases these effects, rewarding only absolute talent and performance. In this global and competitive environment, entry and success are determined by measurable ability rather than arbitrary developmental advantages, underscoring the talent- and performance-driven nature of elite sport labor markets. For youth development systems, reducing relative age biases and supporting late-born athletes can help minimize inefficiencies and talent loss in the broader sport economy.

## Data Availability

The original contributions presented in the study are included in the article/Supplementary Material, further inquiries can be directed to the corresponding author.
